# Identification of circulatory microRNA based biomarkers for early pregnancy diagnosis in buffalo

**DOI:** 10.3389/fcell.2024.1386241

**Published:** 2024-05-06

**Authors:** Kanisht Batra, Anju Sehrawat, Aman Kumar, Man Singh, Ramandeep Kaur, Dipin Chander Yadav, Neha Singh, Sushila Maan

**Affiliations:** ^1^ Department of Animal Biotechnology, Lala Lajpat Rai University of Veterinary and Animal Sciences, Hisar, Haryana, India; ^2^ Department of Livestock Production and Management, Lala Lajpat Rai University of Veterinary and Animal Sciences, Hisar, Haryana, India

**Keywords:** circulatory miRNAs, pregnancy, biomarker, buffalo, implantation

## Abstract

**Introduction:** The most crucial factor in improving animal reproduction efficiency is early pregnancy diagnosis. Early diagnosis not only reduces the time interval between two calvings but also aids farmers in identifying open animals, thereby preventing significant milk production losses. Therefore, the objective of this study was to discover circulatory miRNAs that would be useful for early pregnancy diagnosis in buffalo.

**Material and methods:** Blood samples were taken on 0, 6^th^, 12^th^, and 18^th^ day after artificial insemination from pregnant animals (n = 30) and non-pregnant animals (n = 20). During these stages of pregnancy, total RNA was extracted, and a small RNA library was subsequently generated and sequenced on the Illumina platform. Subsequently, Real-time PCR was used to validate the findings.

**Results and discussion:** There were 4,022 miRNAs found during the pregnancy, with 15 of those lacking sequences and 4,007 having sequences already in the database. From the beginning of pregnancy until the 18^th^ day, 25 of these miRNAs showed a substantial shift in expression levels in the maternal blood, with a change more than two logs. Furthermore, based on qPCR results, 19 miRNAs were found to be more abundant in pregnant animals than in non-pregnant animals. We used target prediction analysis to learn how maternally expressed miRNAs relate to fetal-maternal communication. In conclusion, miRNA based biomarkers that could be associated with the diagnosis of pregnancy were identified including miR-181a and miR-486 highly upregulated on the 18^th^ day of pregnancy. This study also provides a comprehensive profile of the entire miRNA population in maternal buffalo blood during the early stages of pregnancy.

## 1 Introduction

Pregnancy is an essential component of the bovine life cycle that starts with the union of oocytes and sperm until the complete maturation of the fetus. Milk production from animals is solely dependent on the regular pregnancy status of the animal. Bovine fertility rates are low even with high rates of artificial insemination. With 303.76 millions bovines (including buffaloes), India has the biggest population and is the top producer of milk in the world (DAHD, GOI). Although buffaloes are more resistant and more adapted to the Indian climate, their reproductive efficiency is low. This species has several problems, such as delayed puberty, the absence of behavioral estrus, and prolonged *postpartum* ovarian quiescence (de Carvalho et al., 2016). These issues alone account for an estimated 20 million tonnes of milk production loss each year (Petrocchi Jasinski et al., 2023). To maximize reproductive efficiency, it is necessary to develop methods for early pregnancy detection. Rectal organ palpation and signs of an animal such as not returning to estrus are the two most common ways to determine the pregnancy status of the animal. It might be deceptive for farmers when pregnant animals do not exhibit behavioural estrus. Moreover, per-rectal examination may result in mortality during the early embryonic days of buffaloes. Therefore, a detection method at the early stages of pregnancy before 21 days post-artificial insemination is currently needed for buffaloes.

The hormonal or protein-based progesterone assay, the early conception factor assay, the estrone sulphate assay, the interferon-tau stimulated proteins, and the pregnancy-associated glycoproteins are some of the methods for pregnancy detection (Batra et al., 2018a; Batra et al., 2018b; Barile et al., 2021). However, the inherent limitations of these tests make them unreliable for early pregnancy diagnosis. Moreover, most of the current methods only work well after 21 days following insemination and are not very effective in the first 20 days. Researchers now have additional DNA/RNA-based chances to examine biomarker molecules that can assist in bovine pregnancy detection due to the advent of recent molecular tools like next-generation sequencing.

Significant physiological changes in the dam during the first trimester of pregnancy allow for the establishment of cross-talk between the developing embryo and the uterine endothelium, making these early stages of pregnancy pivotal. These alterations lead to the formation of the placenta, which helps nourish the growing calf during the whole gestational period. The placenta in ruminants is a cotyledonary type, which allows signaling molecules to be sent between the fetal cotyledons and the caruncles. A large number of biological processes, including cell differentiation, gene expression, proliferation, and cell death, are regulated by miRNAs, which are small non-coding RNAs that typically consist of 18–22 nucleotides (Wang et al., 2012). These miRNAs significantly affect the maturation of gametes and embryos in both humans and cattle (Legare et al., 2022; Paulson et al., 2022). These miRNAs are necessary for the bovine implantation process (Balaguer et al., 2019; Reza et al., 2019). Moreover, miRNAs play a pivotal role in the initial phases of the uterine wall–embryo communication (Gross et al., 2017; Battaglia et al., 2019). These miRNAs can easily penetrate the peripheral circulation of dams during the initial stages of pregnancy. During pregnancy and the estrous cycles of cattle, certain miRNAs have been found to be in circulation (de Ávila et al., 2020). It is worth noting that no research has yet identified or discovered the miRNA repertoire in maternal plasma during the early stages of pregnancy in buffaloes. The purpose of evaluating miRNA expression as a biomarker for early pregnancy detection in buffalo was to gain insight into the aforementioned scenario.

## 2 Materials and methods

The intended research would focus on several miRNAs present in mature form and in circulatory exosomes in buffalo maternal blood. To prepare the libraries for sequencing, these miRNAs were isolated from blood samples and a small miRNA library preparation kit was utilized. Total small RNA sequencing was used to find the miRNAs that have differential levels of expression in the blood of pregnant and non-pregnant animals.

### 2.1 Collection of samples

In order to get blood samples for this study, fifty buffalo were chosen from various livestock farms, LUVAS, and different villages in Haryana. This area has a semiarid climate, with very hot summers and relatively cool winters. The collection of samples was done mostly during autumn season having a high conception rate. Blood samples were taken after artificial insemination of animals exhibiting estrus symptoms (0^th^ day). The jugular vein was punctured and blood samples were collected in tubes containing EDTA (Sigma, St. Louis, Missouri, United States) under sterile conditions. Blood samples were obtained from animals on different days such as 0^th^, 6^th^, 12^th^, and 18^th^ day after artificial insemination. The extraction of small RNAs from these samples was performed immediately after collection. Those animals that showed heat behavior and discharge after 21 days were again inseminated, and samples collected from them were kept for control studies. Retrospective studies were conducted on non returning animals on 35^th^ day with an ultrasound machine for visualization of the fetus inside the uterus (Toshiba, Japan). The animals were monitored for an extra three to 10 months to confirm their pregnancy status.

### 2.2 Isolation of small RNAs

Blood samples from pregnant and non-pregnant animals were used for the extraction of miRNAs. The protocol for the extraction of miRNAs was standardized using a Promega miRNA isolation kit (Promega Inc., Madison, Wisconsin, United States) in combination with the TRIzol (Sigma, St. Louis, Missouri, United States) method to obtain the maximum concentration of miRNAs. In a nutshell, the blood cells were completely lysed by adding 1 mL of TRIzol reagent to 600 µL of the blood sample. Adding 200 µL of chloroform (0.2 volume TRIzol reagent) to the mixture allowed for phase separation to occur. The phases were separated by centrifuging the mixture at 12,000 rpm for 15 min at 4°C. Isopropanol was equally mixed with the recovered supernatant for RNA precipitation. The whole homogenate was centrifuged for 1 minute at 12,000 rpm using the ReliaPrepTM minicolumn following the instructions provided by the manufacturer (Promega Inc., Madison, Wisconsin, United States), for isolation of the micro RNAs. Finally, these micro RNAs from the extraction process were eluted in 30 µL nuclease-free water and kept at −80°C for further use. An HS sensitivity broad spectrum RNA assay kit was used to quantify the small RNA extracted from the kit process using a Qubit 2.0 Fluorometer.

### 2.3 Library preparation

The extracted miRNAs were used for cDNA library preparation. In order to compile the library, fifty samples were used, consisting of both pregnant and non-pregnant animals, taken at 0^th^, 6^th^, 12^th^, and 18^th^ day post-AI. The miRNA library was prepared using a TruSeq Small RNA library preparation kit (Illumina, San Diego, CA, United States), with a few minor modifications including ligation and incubation time for generation of cDNA. This was done to ensure the production of the optimal library for every sample. Briefly, the miRNA library was prepared by first ligating 3′adapters to miRNAs. The RA3 (RNA 3′Adapter) combination was mixed with 1 μg of total RNA, and then incubated at 70°C for 2 min. Before incubating the cDNA at 28 °C for an hour, the 3′end was supplemented with 2 μL of HML (ligation buffer), 1 μL of T4 RNA Ligase 2 (M0239S), and 1 μL of RNase Inhibitor. The 5′adapter was then ligated by adding 1.0 μL of RA5 (RNA 5′Adapter) and incubating for 2 min at 70°C. The generated product was combined with 1 μL of 10 mM ATP and 1 μL of T4 RNA ligase for amplification, which was then incubated at 28°C for 1 h. After the adapter-ligated RNA tube was filled with 1 μL of RNA RT Primer, the mixture was incubated at 70°C for 2 min again. For the second strand synthesis, the adapter-ligated RNA/primer mix was mixed with 5X First Strand Buffer, 12.5 mM dNTP Mix, 100 mM DTT, RNase Inhibitor, and SuperScript II Reverse Transcriptase. The adapter-ligated RNA reaction was left to incubate at 50°C for an extra hour. To amplify the library, index primers RP1 and RPIX and PML master mix (25 μL) were utilized. An amplification step was performed by subjecting the reaction to a temperature of 98°C for 30 s, followed by eleven cycles of 60°C for 30 s, 72°C for 15 s, and 72°C for 10 min. The prepared library was checked by agarose gel electrophoresis for cutting of the desired band.

The cDNA library prepared by different protocols of library preparation (Ioannidis and Donadeu, 2016; Ioannidis and Donadeu, 2017; Sarwalia et al., 2021) was analyzed using agarose gel electrophoresis and a fragment analyzer. A 147 bp band was visible on the agarose gel, indicating the presence of mature miRNA. This miRNA was generated from 22 nt short RNA fragments. Some miRNAs, other short regulatory RNAs, and piwi-interacting RNAs make up the other band, which is 157 bp long. To serve as a marker, three dsDNA fragments-145 base pairs, 160 base pairs, and 500 base pairs were included from the kit’s custom resolution ladder. In order to separate and clean the bands located above the 145 bp band and below the 160 bp Custom Resolution Ladder band, a Qiagen gel extraction kit (Qiagen, Germantown Rd, Germantown MD 20874 USA) was employed. A fragment analyzer was used to examine the library. The samples showing proper peak and maximum cluster generation in the proper ranging concentration of the cDNA library were loaded on the next-generation sequencer (MiSeq).

### 2.4 Next-generation sequencing using MiSeq

Blood samples from both pregnant and non-pregnant animals were subjected to complete small RNA sequencing, with the goal of identifying small miRNAs. The MiSeq Reagent Kit v3 was run on a MiSeq next-generation sequencer, which contains ready-to-use reagent cartridges, and the cluster density, cycle time and good quality (Q) scores were optimized.

### 2.5 Bioinformatic analysis

The FastQC tool version 0.11.51 was used to visualize the quality of the raw miRNA data, revealing adapters and several reads with subpar Phred scores (Andrews, 2010). The 30 Illumina adapters were removed using Trimgalore 0.6.5. Lastly, the filtered reads were aligned with the buffalo genome Ensembl UMD3.1 using Bowtie2. For expression data normalization in NGS pipeline, TMM (The Trimmed Mean of M-values) was used. It was employed to adjust for differences in sequencing depth and other systematic biases across samples, ensuring that the expression levels of genes are comparable between samples. Different technical replicates were also included in this study. The novel bovine miRNA, which is only found in the maternal blood of pregnant animals, was discovered through the analysis of raw sequencing data. miRDeep2 was used for the discovery of novel miRNA from raw sequence data. It employs a machine learning approach to predict novel miRNAs based on characteristic features of miRNA biogenesis and sequence conservation. The smrnaseq pipeline was used in conjunction with NF-CORE version 2.3 to identify miRNAs. Filtering parameters for the differential expression study of miRNA were a *p*-value of 0.05 and the log-fold change in gene expression levels. The iGEAK programme was used to detect miRNAs that were significantly differently expressed. miRNAs were deemed to be regularly expressed if their fold change values were less than two and more than −2.

### 2.6 Real-time expression analysis

To further confirm the expression levels of the selected miRNAs, real-time PCR based on SYBR-based chemistry was employed. The U6 miRNA (Housekeeping miRNA) was utilized as a control miRNA. The Real-time expression analysis was focused on the relative expression level of the 25 most prevalent miRNAs identified by next-generation sequencing (NGS) investigation. The cDNAs for the miRNAs were synthesized using a Qiagen miScript cDNA Synthesis kit, as directed by the manufacturer. To summarise, 5 μg of pure miRNA was utilised to generate cDNA using 20 μL reactions, which included 4 μL of 5x miScript HiSpec Buffer, 2 μL of 10x miScript Nucleics Mix, and 2 μL of miScript Reverse Transcriptase Mix. The generated primer/template mixture was incubated for 60 min at 37°C before being inactivated for 5 min at 95°C. The specific primers for these miRNAs were designed using the miRPrimer tool using conserved sequences present in the miRNA database. To get miRNA sequences that were not already present, we first extracted them from the NGS analysis sequences and then substituted thymine with uracil. For amplification of cDNA prepared, QuantiTect SYBR Green PCR Master Mix was used with ABI step one real-time PCR equipment. A 20 µL reaction containing 10 µL of 2x QuantiTect SYBR Green PCR Master Mix, 2 µL of 10x miScript universal primer, and 2 µL of 10x miScript primer specifically made for each miRNA was used to optimize the real-time PCR assay. Three biological replicates were used as templates for the analysis of all samples.

### 2.7 Analysis of real-time expression

Statistical analysis of miRNA concentrations in several blood samples was performed to detect early pregnancy in buffaloes. The average Ct value fold change was determined using the results of Schmittgen and Livak (2008). A one-way analysis of variance (ANOVA) was used for the statistical analysis, and a *p*-value below 0.05 was deemed highly significant. To evaluate the discriminating power of each miRNA for pregnancy detection, receiver operating characteristic (ROC) curves were constructed. The area under the ROC curve (AUC), which provides a numerical measure of the test’s accuracy, was calculated as the sum of the areas of the trapezoids.

### 2.8 *Insilico* prediction of miRNAs Target

To find potential mRNA targets that are unique to the miRNAs with differential expression, miRNAcons target software was used, which is based on input data provided by Miranda, PITA and TargetSpy for animal miRNAs. Target scan search engines and the miRNA Path DB were used to predict the targeted pathways.

## 3 Results

### 3.1 Sequencing of maternal blood on different days of pregnancy

**FIGURE 1:**
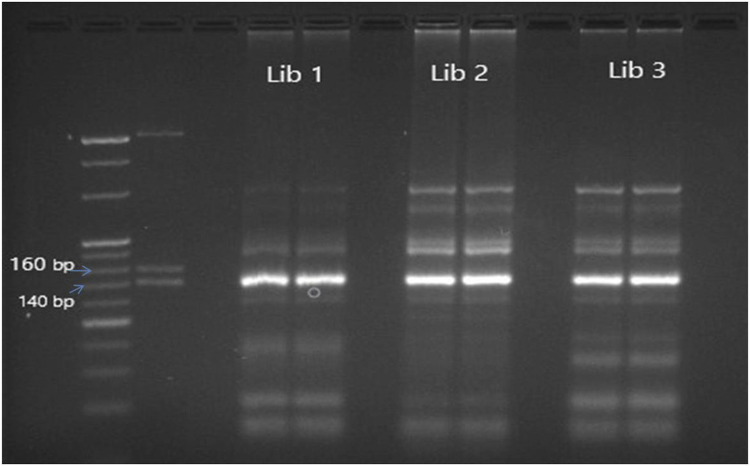
Agarose gel electrophoresis of unpurified miRNA libraries prepared using Trueseq library preparation kit depicting library size of 147–157 bp. L1,L2,L3: Library prepared from different samples.

**FIGURE 2:**
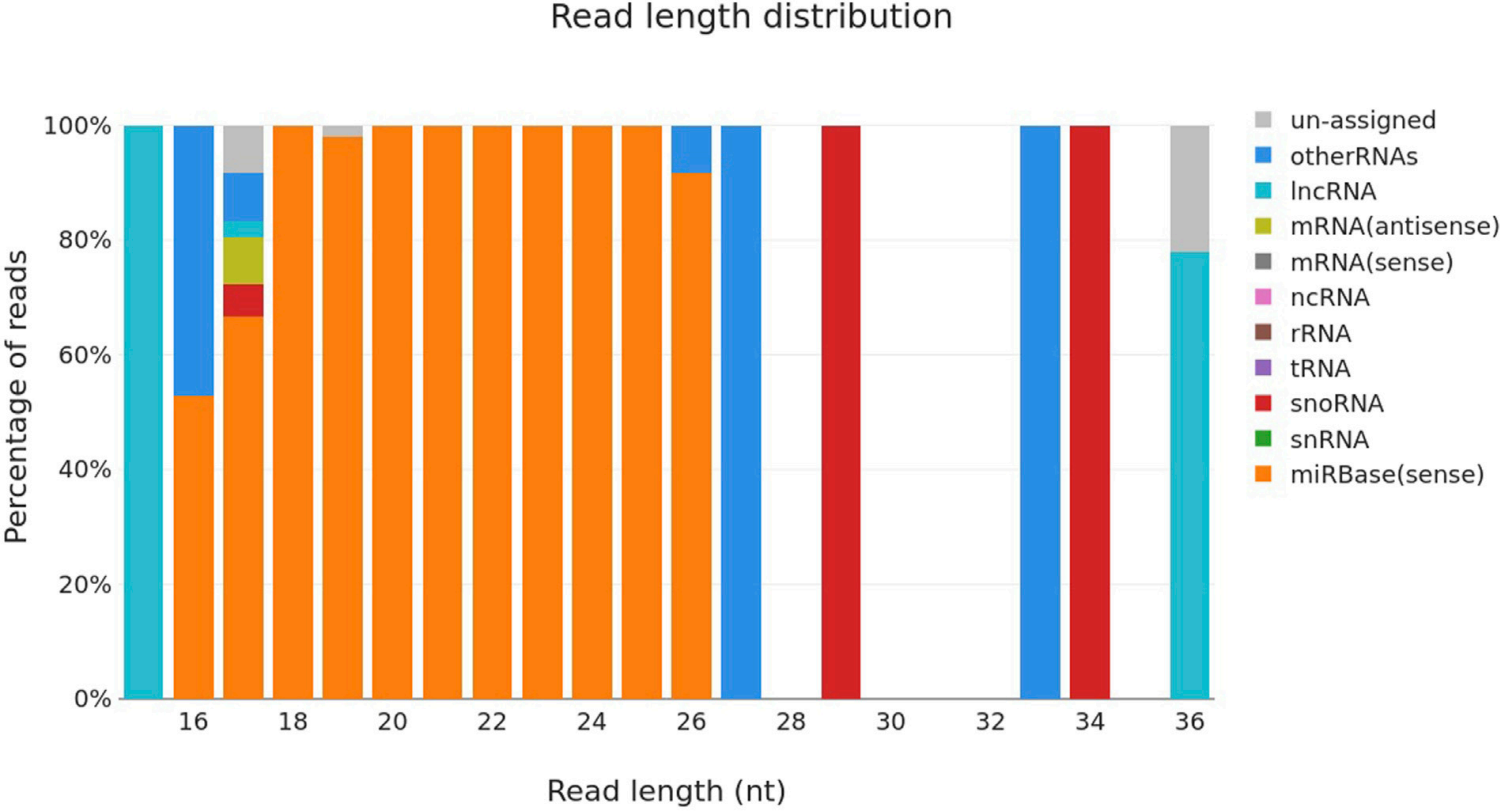
Read length distribution curve of different types of RNA sequenced using Miseq system.

There were a total of n = 30 animals that were pregnant and n = 20 animals were truly non-pregnant. A total of n = 50 small RNA libraries were prepared for next-generation sequencing of samples. The samples were collected at several points throughout the pregnancy, namely, on the 0^th^, 6^th^, 12^th^, and 18^th^ day a time that is generally considered ideal for pregnant molecules. Sequencing was performed using the bands depicted in Figure 1, which include mature miRNA and were located at the top of the 145 bp and below the 160 bp custom resolution ladder. The bulk of the reads were mature miRNA, according to the length distribution curve that was displayed following adaptor cutting (Figure 2). For every sample, there were about 2,164,224 raw input reads and 29,208 adapter trimmed reads. The percentage of adapter dimers was only 0.04%. There were 99.8% reads that mapped to miRNAs, 5.36% that were detected as hairpin miR, 5.73% that were detected as mature miR and 0.05% that were mapped to miRBase hairpins.

### 3.2 Identification of the different miRNAs in maternal blood on different days of pregnancy

**FIGURE 3:**
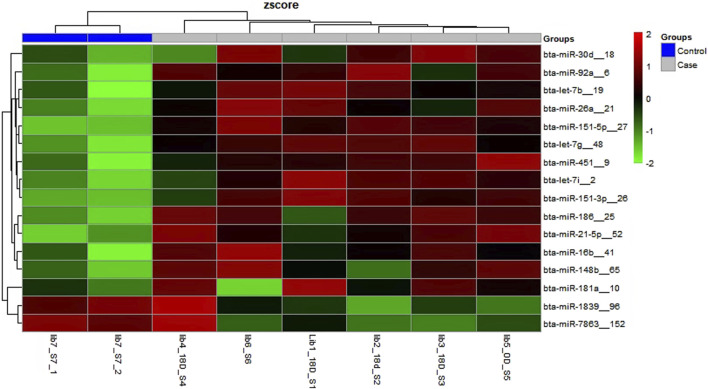
Heatmap of the miRNAs that had significant expression levels in the early days of pregnancy.

**FIGURE 4:**
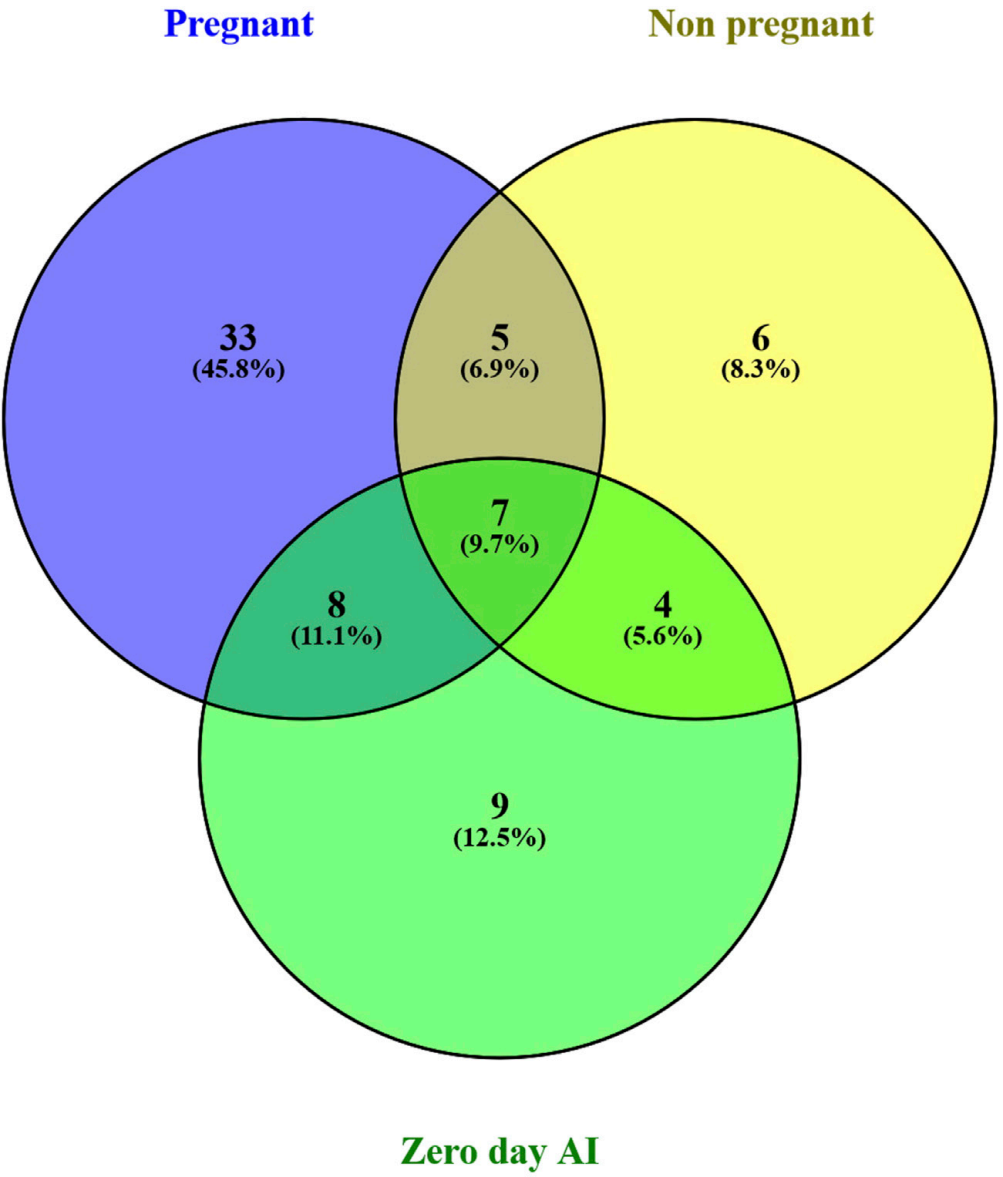
Venn diagram of the miRNAs population in early days of pregnancy.

There were a total of 4,022 distinct miRNAs that were detected on various days throughout pregnancy. The miRBase database of miRNAs had 4,007 miRNAs, whereas the remaining 15 were completely new and did not have any sequences already. We created a heatmap and venn diagram to display the miRNAs with the highest expression levels throughout the first trimester of pregnancy (Figures 3, 4). There was significant shift in maternal blood expression of 25 miRNAs between the 0^th^ and day 18^th^ day of pregnancy. During the early stages of pregnancy, a several miRNAs (miRNAs) exhibited notable upregulation, including miR-181a, miR-423-3p, miR-let-7f, miR-21-5p, miR-127, miR-141, miR-146b, miR-191, miR-31, miR-223, miR-10a, miR130b, miR-155, miR 205, miR-2284v, miR-101, miR-665, miR-379, miR-152, miR-2483, miR-10b, miR-146a, miR-143, miR-103, and bta-miR-486. There were 154 other miRNAs that were downregulated on different days of pregnancy. On the 6^th^ and 12^th^ days, miR-191, miR-127, let-7d, miR-451, let-7i, and miR-151-3p revealed increased expression levels in pregnant animals, but they were less than twofold. The summarizations of miRNAs show upregulation in pregnant animals on 18^th^ day of pregnancy are given in Table 1 miR-141, miR-223, let-7f, miR-423-3p, miR-146b, miR-101, miR-31, and miR-148b were reported in abundance in pregnant animals compared to non-pregnant animals, but the fold change increase was less than twofold. There were 55 miRNAs that were upregulated by less than one-fold. Out of all miRNAs, miR-181a and miR-486 were reported in high numbers and have threefold increase in expression during first 18 days of pregnancy.

**TABLE 1:** Different microRNAs reveal differential expression changes identified by small RNA sequencing.

Sr no	Micro RNAs	Fold changes as compared to NP	*p*-Value
1.	bta-miR-181a	4.79	1.26E-03
2.	bta-miR-21-5p	2.87	1.50E-03
3.	bta-let-7i	1.00	2.41E-03
4.	bta-miR-101	0.93	1.33E-02
5.	bta-miR-148b	1.75	3.37E-02
6.	bta-let-7f	1.30	3.75E-02
7.	bta-miR-16a	0.41	5.62E-02
8.	SNORD29	0.60	5.91E-02
9.	bta-miR-152	1.24	6.24E-02
10.	bta-miR-2483	1.44	6.33E-02
11.	bta-miR-143	1.34	6.45E-02
12.	bta-miR-151-3p	1.04	6.54E-02
13.	bta-miR-127	1.49	6.90E-02
14.	bta-miR-141	0.73	1.66E-02
15.	bta-miR-342	1.50	9.46E-02
16.	bta-miR-186	0.95	1.71E-01
17.	bta-miR-99b	0.86	1.75E-01
18.	bta-miR-27b	1.74	1.81E-01
19.	bta-miR-486	4.14	6.46E-01
20.	bta-miR-150	0.17	1.86E-01
21.	bta-miR-92a	0.23	2.08E-01
22.	bta-miR-423-3p	0.59	3.81E-01
23.	bta-miR-191	0.88	3.97E-01
24.	bta-let-7d	1.95	4.02E-01
25.	bta-miR-10b	0.39	4.11E-01
26.	bta-miR-19b	2.30	4.19E-01
27.	bta-miR-93	0.46	4.20E-01
28.	bta-miR-15b	0.73	4.22E-01
29.	bta-miR-10a	0.67	4.22E-01
30.	bta-miR-769	1.64	4.69E-01
31.	bta-miR-1271	1.96	4.71E-01
32.	bta-let-7g	0.32	4.84E-01
33.	bta-miR-155	1.91	4.85E-01
34.	bta-miR-192	0.45	4.96E-01
35.	bta-miR-205	0.52	5.76E-01
36.	bta-miR-379	0.78	5.90E-01
37.	bta-miR-151-5p	0.49	6.23E-01
38.	bta-miR-98	1.43	6.28E-01
39.	bta-miR-425-5p	0.48	6.34E-01
40.	bta-miR-223	0.56	6.57E-01
41.	bta-miR-6119-5p	0.46	6.77E-01
42.	bta-miR-16b	1.53	6.92E-01
43.	bta-miR-451	0.42	7.06E-01
44.	bta-miR-2284	0.34	7.56E-01
45.	bta-miR-130b	1.31	8.12E-01
46.	snoMe28S-Am982	1.50	8.20E-01
47.	bta-miR-146b	0.43	8.22E-01
48.	bta-miR-30a-5p	0.55	8.26E-01
49.	bta-miR-339a	1.18	8.82E-01
50.	bta-miR-31	0.58	8.86E-01
51.	SNORD43	0.54	8.89E-01
52.	bta-miR-23b-3p	0.06	8.96E-01
53.	bta-miR-374a	1.06	9.03E-01
54.	bta-miR-423-5p	0.08	9.03E-01
55.	bta-miR-146a	1.20	9.12E-01
56.	bta-miR-652	0.01	9.17E-01
57.	bta-miR-103	0.09	9.24E-01
58.	bta-miR-30b-5p	0.27	9.36E-01
59.	bta-miR-320a	0.59	9.42E-01
60.	SNORD48	0.27	9.47E-01
61.	bta-miR-345-3p	0.86	9.48E-01
62.	bta-miR-421	1.00	9.63E-01
63.	bta-miR-7	1.15	9.68E-01
64.	bta-miR-665	0.77	1.00E+00

### 3.3 RT‒qPCR validation

**FIGURE 5:**
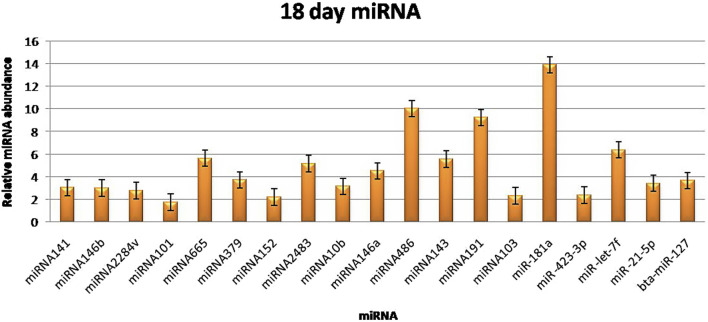
Graphical representation of the abundance of miRNAs in the blood of pregnant and non-pregnant animals on 18^th^ day. Here, *X*-axis represent the relative miRNA abundance and *Y*-axis represent the Days.

**FIGURE 6:**
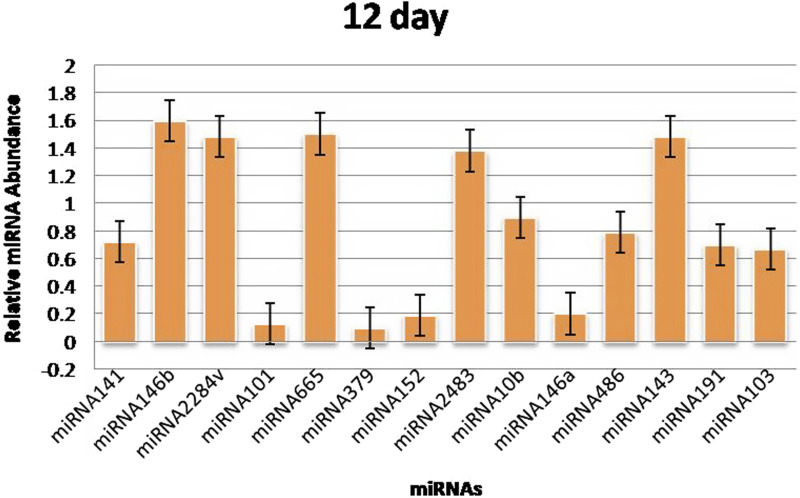
Graphical representation of the abundance of miRNAs in the blood of pregnant and non-pregnant animals on 12^th^ day. Here, *X*-axis represent the relative miRNA abundance and *Y*-axis represent the Days.

**FIGURE 7:**
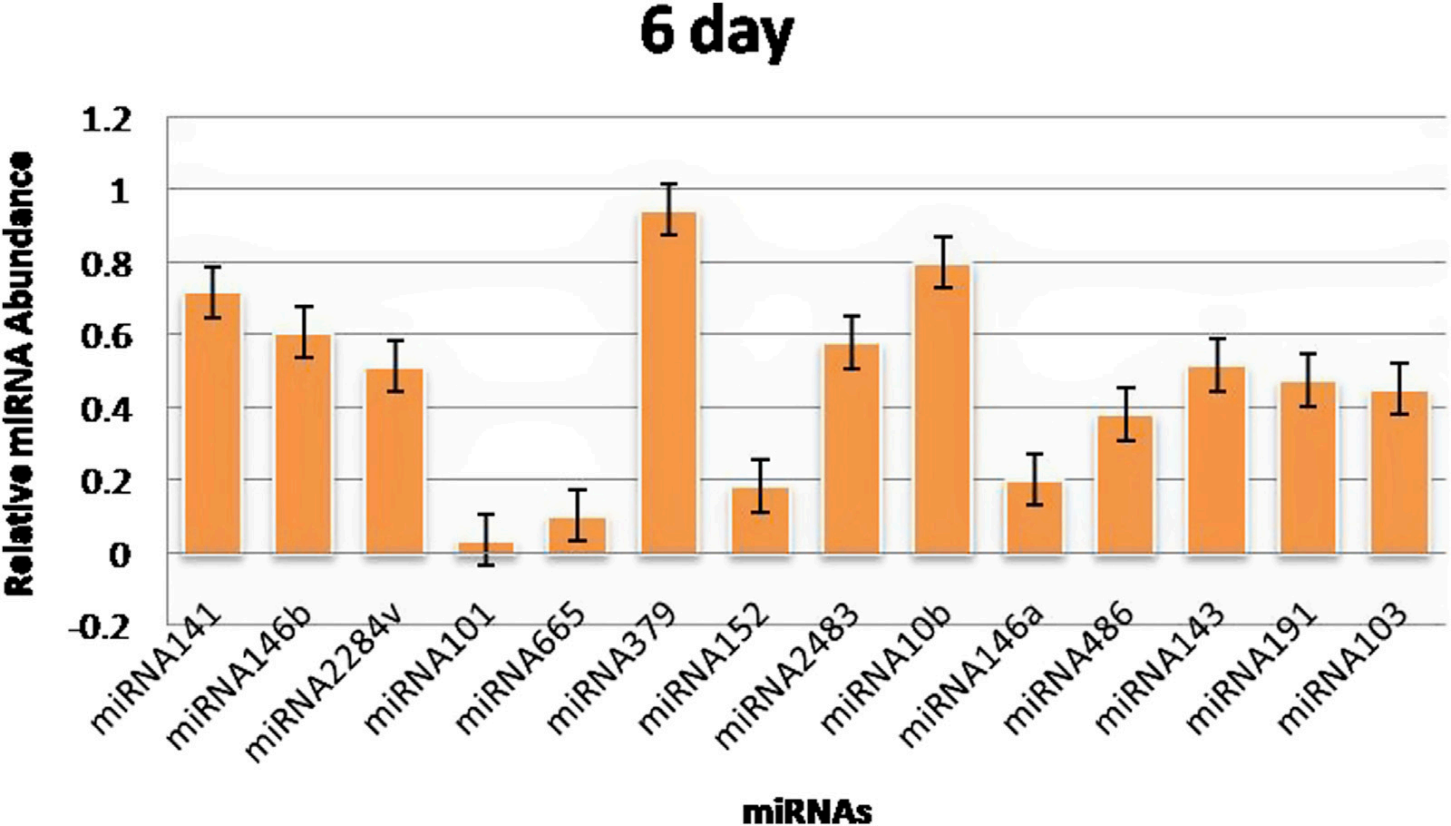
Graphical representation of the abundance of miRNAs in the blood of pregnant and non-pregnant animals on 6^th^ day. Here, *X*-axis represent the relative miRNA abundance and *Y*-axis represent the Days.

The RT‒qPCR test was optimized at 40 cycles of initial denaturation at 95°C for 15 min, denaturation at 94°C for 15 s, annealing at 55°C for 30 s, and amplification at 70°C for 30 s. For validation using qPCR, 25 miRNAs with significant expression were shortlisted that showed differences between pregnant and non-pregnant animals. The duration between 18 and 20 days is considered to be significant because it involves implantation; therefore, miRNAs with more expression during the 18^th^ day were selected. Nineteen miRNAs exhibited a higher concentration in the pregnant group on the 18^th^ day of pregnancy than in the non-pregnant group. On the 0^th^, 6^th^, 12^th^, and 18^th^ days of artificial insemination, the mean cycle threshold values for miRNAs were computed (Table 2). miR-141, miR-146b, miR-2284v, miR-101, miR-665, miR-379, miR-152, miR-2483, miR-10b, miR-146a, miR-486, miR-143, miR-191, miR-103, miR-181a, miR -423-3p, miR -let-7f, miR -21-5p, and miR-127 were significantly upregulated in the pregnant animals on 18^th^ day (Figure 5). Furthermore, the expression of miR-146b, miR-2284v, miR-665, miR-2483 and miR-143 was upregulated on the 12^th^ day in pregnant animals compared to non-pregnant animals (Figure 6). The expression of miR-141, miR-379, miR-10b, miR-2483 and miR-146b was upregulated on the 6^th^ day in pregnant animals compared to non-pregnant animals (Figure 7).

**TABLE 2:** Relative quantitation expression analysis of the miRNAs with reference to the endogenous reference miRNA.

miRNAs	Cт mean (P)	Cт mean (NP)	Cт SD	ΔΔCт mean	RQ
miRNA141	25.166^a^	26.784	0.078	−1.618	3.05
miRNA146b	22.66^a^	24.257	0.012	−1.597	3.01
miRNA2284v	26.88^a^	28.364	0.014	−1.484	2.78
miRNA101	21.99^b^	22.818	0.09	−0.828	1.76
miRNA665	18.66^a^	21.16	0.04	−2.50	5.65
miRNA379	16.99^a^	18.89	0.12	−1.900	3.73
miRNA152	10.186 ^b^	11.34	0.015	−1.154	2.21
miRNA2483	9.609^a^	11.988	0.118	−2.379	5.169
miRNA10b	8.645 ^b^	10.313	0.090	−1.668	3.16
miRNA146a	16.99 ^b^	19.175	0.02	−2.185	4.53
miRNA486	15.88^a^	19.216	0.15	−3.336	10.05
miRNA143	23.99^a^	26.471	0.04	−2.481	5.57
miRNA191	14.88^a^	18.097	0.07	−3.217	9.25
miRNA103	19.88 ^b^	21.11	0.015	−1.230	2.34
miR-181a	13.66^a^	17.536	0.07	−3.876	13.92
miR -423-3p	21.55 ^b^	22.804	0.033	−1.254	2.378
miR -let-7f	25.88^a^	28.558	0.077	−2.678	6.399
miR -21-5p	27.88^a^	29.669	0.073	−1.789	3.434
bta-miR-127	15.68^a^	17.569	0.014	−1.889	3.68

^a,b,c^Mean values bearing different superscripts in a column differ significantly (*p* < 0.05).

### 3.4 Target prediction of miRNAs *in silico*

**FIGURE 8:**
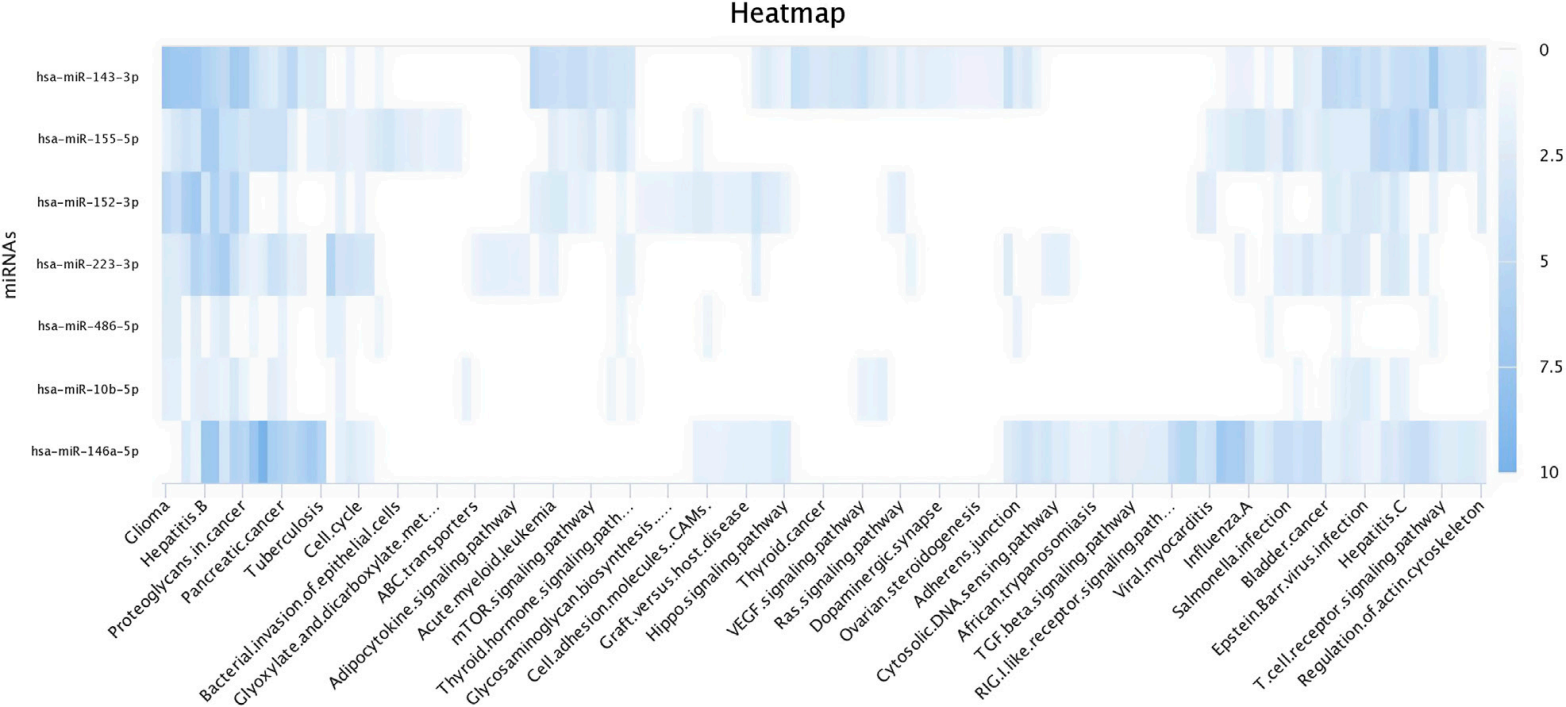
Heat map for Gene Ontology categories of differentially expressed known microRNA first seven for different biological processes.

**FIGURE 9:**
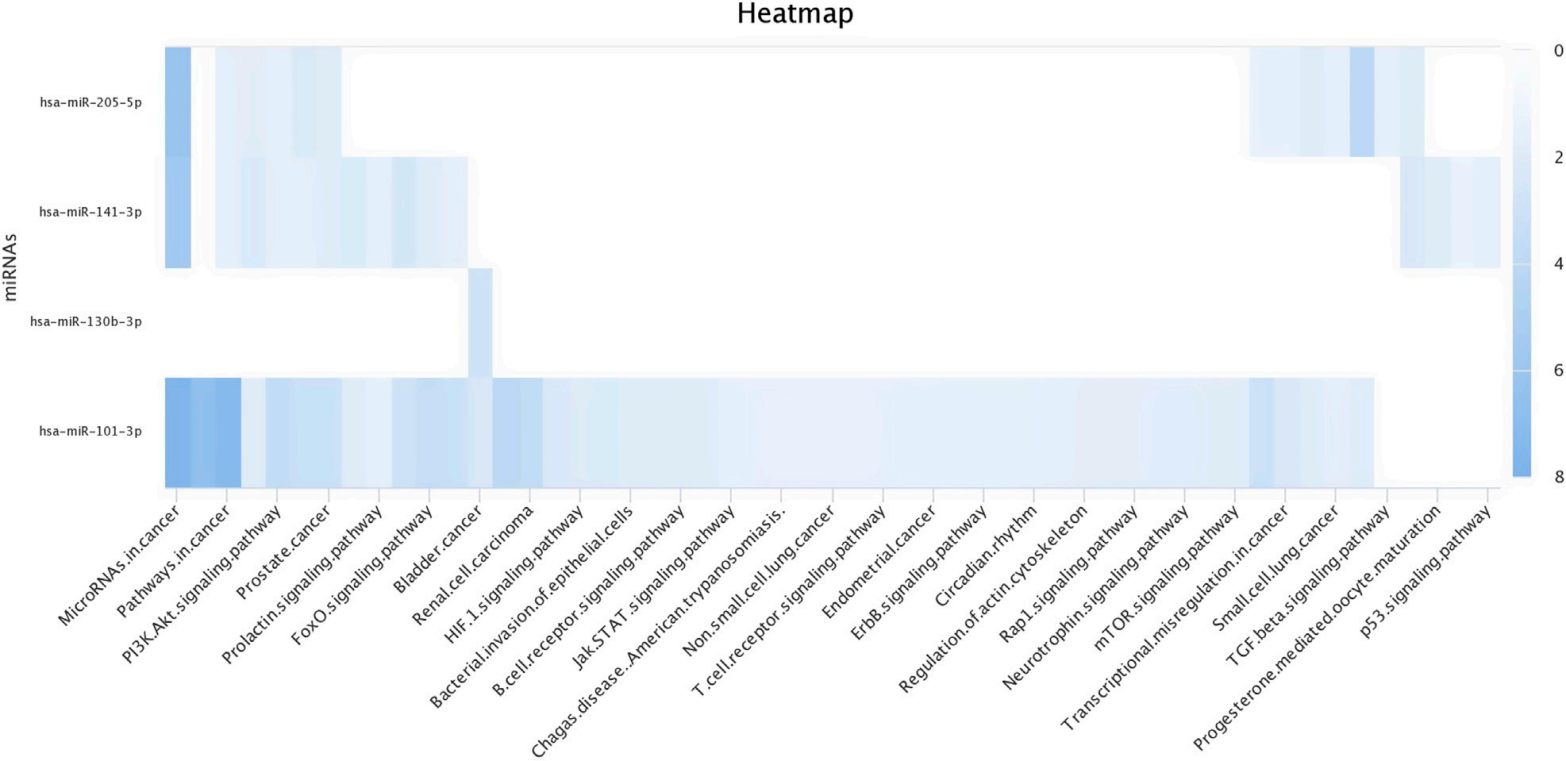
Heat map for Gene Ontology categories of differentially expressed known microRNA another four for different biological processes.

**FIGURE 10:**
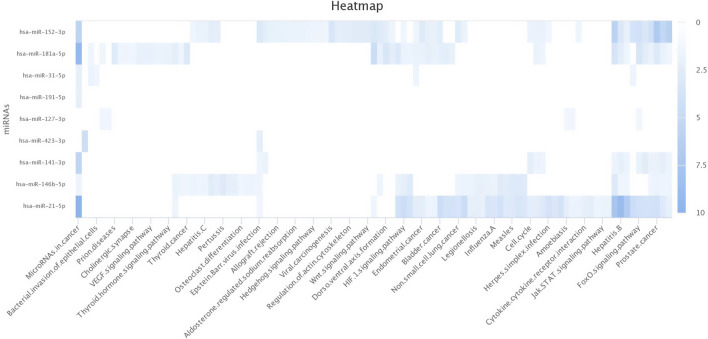
Heat map for Gene Ontology categories of differentially expressed known microRNA another nine for different biological processes.

The top upregulated miR predicted genes used for target prediction were miR-181a, miR-146b, miR-31, miR-486, miR-223, miR-125a, miR-21-5p, miR-10a, miR130b, miR-155, and miR-205. To determine the biological roles of the targeted genes, assessments of the Kyoto Encyclopedia of Genes and Genomes (KEGG) pathway enrichment and Gene Ontology (GO) function were carried out (Figures 8–10). The top most targeted genes were toll like receptor 4 (TLR4), Fos proto-oncogene, AP-1 transcription factor subunit (FOS), LIF interleukin six family cytokine (LIF), suppressor of cytokine signaling 3(SOCS3), cytoskeleton associated protein 4(CKAP4), integrin subunit alpha V(ITGAV), fibroblast growth factor 7(FGF7), myocyte enhancer factor 2C (MEF2C), interleukin six (interferon, beta 2) (LOC517016), mitogen-activated protein kinase kinase kinase 11 (MAP3K11), fibrinogen like 2(FGL2), arginase 2 (ARG2), SNAP associated protein (SNAPIN) paternally expressed 3(PEG3), zinc finger and BTB domain containing 38 (ZBTB38) (Additional file 1).

### 3.5 Statistical analysis

**FIGURE 11:**
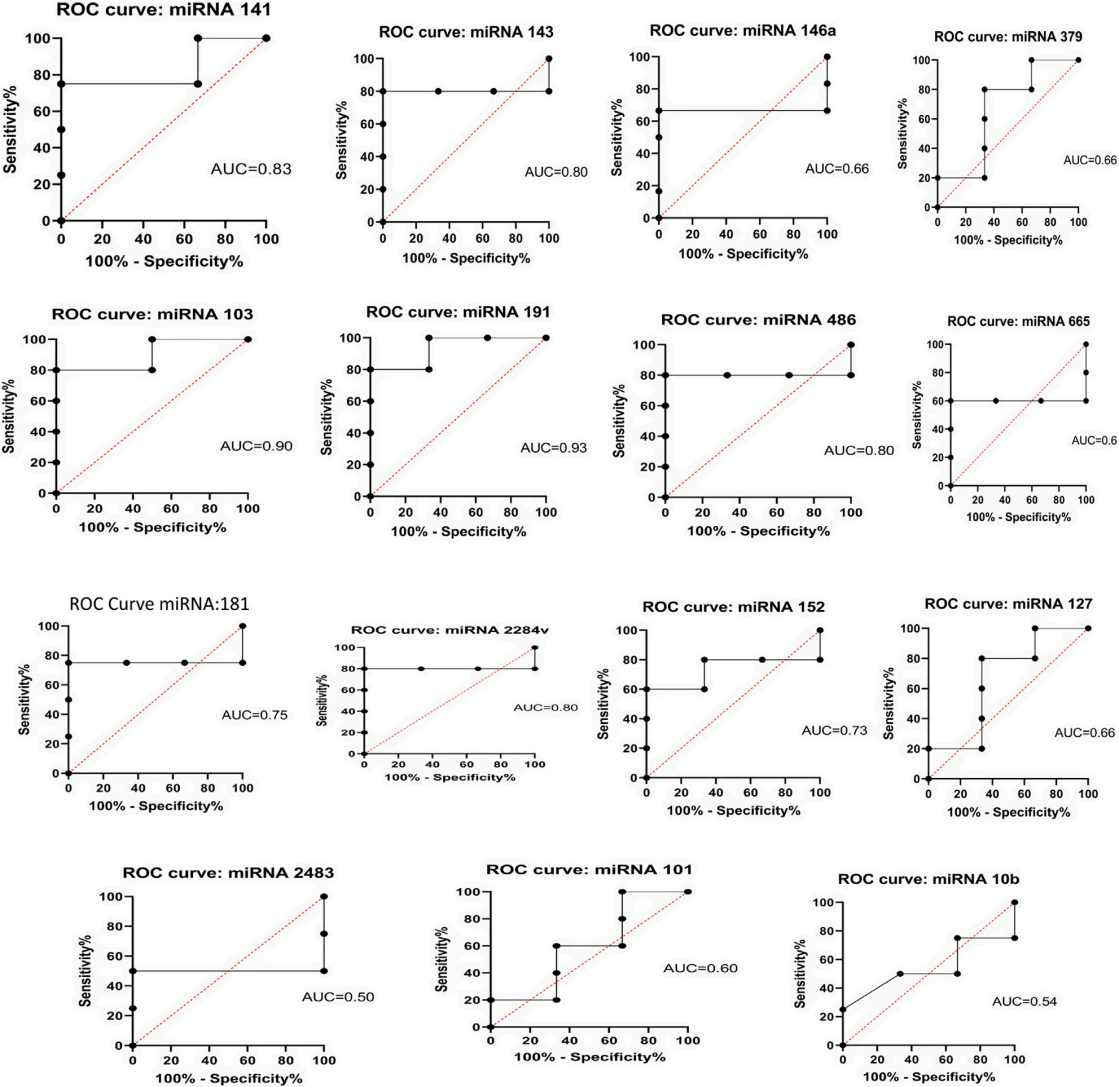
ROC curve analysis of diagnostic value of circulating miRNA for pregnant vs. non-pregnant buffaloes: AUC, area under the curve; ROC, receiver operating characteristic.

There were 19 miRNAs on the 18^th^ day of pregnancy that showed a higher concentration in the pregnant group than in the non-pregnant group. A receiver operating characteristic (ROC) curve was generated for each miRNA that showed upregulation during pregnancy to determine the diagnostic value of each miRNA. miRNAs with an AUC >0.7 served as effective biomarkers for identifying pregnant animals. Statistical analysis using one-way ANOVA (*p* < 0.01) showed that there were notable differences among the pregnant group at both time periods. The AUC for these miRNAs was specifically 60%–80% (Figure 11). To ascertain whether the test was capable of differentiating between pregnant and non-pregnant animals, the ROC curve was analyzed by testing the null hypothesis that the AUC = 0.50.

## 4 Discussion

Deciphering the precise miRNAs that can serve as biomarkers for early pregnancy detection in dairy animals is a current focus of numerous laboratories. Evidence of the existence of pregnancy-specific miRNAs and their role as potential markers in pregnancy diagnosis has recently been published in many journals (Ioannidis and Donadeu, 2016; 2017; Sarwalia et al., 2021). The microRNA profiling in bovine at 8 weeks has been described for comparative studies (Lim et al., 2021). The miRNAs expression levels from intrauterine exosomes have been analyzed in cows 7 days after fertilization. There were twenty two miRNAs which were differentially expressed in pregnant animals on the 7^th^ day. (Zhai et al., 2022).

Individually, the bta-mir 140 has been discovered as a viable prospective miRNA with a role as a biomarker for early pregnancy detection (Fiandanese et al., 2015). This miRNA has been reported to be upregulated in all pregnant cows on the 19^th^ day after artificial insemination and was upregulated in pregnant, nonlactating cows from the 13^th^ day onwards. In a similar study, six different miRNAs (bta-miR-26a, bta-miR-29c, bta-miR-138, bta-miR-204, bta-miR-1249 and hsa-miR-4532) were also identified that were differentially expressed on different days of pregnancy in heifers (Ioannidis and Donadeu, 2016; 2017). In both pregnant and non-pregnant cows as well as cows experiencing early embryonic death, exosomal miR-s are variably expressed (Pohler et al., 2017). Serum from pregnant cows shows differential expression of miRNA-8 on 19^th^ day and miRNA-23 on 24^th^ day (Gebremedhn et al., 2018). Similarly, 29 miRs exhibited significant expression in dairy cattle on 30^th^ day of pregnancy (Markkandan et al., 2018). Therefore, in view of the above background, the miRNAs were identified having the capability of detecting different days of pregnancy in buffalo.

Expression profiling using the NGS technique provided a complete dataset for the miRNA transcriptome present in the blood of pregnant animals on different days of pregnancy. Using the sushila maan RNA seq pipeline, it was found that millions of miRNAs are abundant in the blood of animals. There are many reports related to the abundance of the miRNA transcriptome in cattle and buffalo using the NGS platform (Guelfi et al., 2017; Sarwalia et al., 2021; Ono et al., 2022). However, none of the reports have deciphered the transcriptome of miRNAs in the blood of buffalo during different days of pregnancy. These miRNAs in buffalo were characterized in the present study based on different days of pregnancy using NGS technology. There were more than 25 miRNAs that showed significant differences (log fold change 2, *p* < 0.05) in NGS analysis. The NGS results were revalidated by qPCR which also indicated significant upregulation of these miRNAs in the blood of pregnant animals on different days of pregnancy. In another study, 16 miRNAs were significantly upregulated in the plasma of pregnant animals (Ioannidis and Donadeu, 2016). miR-26a and miR-1249 were significantly upregulated on different days of pregnancy, as shown by RT‒qPCR validation in abovestudy. In the present study, the miRNAs with significant differences were validated by qPCR to determine their ability as biomarkers for differentiating pregnant animals from non-pregnant animals.

In particular, the miRNAs that showed significant upregulation were bta-miR-181a, bta-miR-423-3p, bta-miR-let-7f, bta-miR-21-5p, bta-miR-127, bta-miR-141, bta-miR-146b, bta-miR-191 and bta-miR-486 in the early stages of pregnancy. The expression level of miR-181a was shown to rise significantly in human endometrial stromal cells treated with 8-Br-cAMP and MPA (Zhang et al., 2015). The expression of genes linked to human endometrial stromal cell decidualization and morphological transformation is encouraged by the elevated concentration of miR-181a. KLF12 is downregulated at both the transcriptional and translational levels as a result of miR-181a’s interaction with the transcription factor’s 3′untranslated region. Decidualization caused by miR-181a was eliminated by KLF12 overexpression. Additionally, let-7 g-5p, 7f, and 7i have been linked to endometrial decidualization and may have a role in the control of pregnancy (Zhao et al., 2021). As a result, it is possible to examine let-7 g-5p, 7f, and 7i as prospective diagnostic biomarkers for predicting the response of the female ovary to stimulation. Similarly, during the early stages of pregnancy, we noticed enhanced expression of bta-miR-let-7f. It has also been noted that hsa-miR-191-5p is strongly expressed in the culture media of embryos that result in pregnancy (*p* ≤ 0.001) (Acuña-González et al., 2021). This demonstrates the significant expression of miR-191 during the implantation phase and provides reliable early pregnancy indications.

In the third trimester, trophoblast cells express the pregnancy-related miR-141 at higher levels in maternal plasma (Ospina-Prieto et al., 2016). Along with its role in intercellular communication between foetal trophoblasts and maternal immune cells, miR-141 regulates function of trophoblasts and immune cells. It is inappropriately expressed in preeclampsia (PE) placentas. The function of exosomal miR-486-5p, which originates from human placental cells, is crucial for the migration, proliferation, and invasion of trophoblast cells (Ma et al., 2021). These results also show that miR-141 and 486-5p are highly expressed during the implantation phase, which means that these molecules can be used as accurate markers of early pregnancy.

The levels of bta-miR-146b and bta-miR-27b are elevated in the immunological response, preeclampsia, and mammary gland development (Markkandan et al., 2018). The maternal placenta has been found to contain increased levels of miR-423-3p, bta-miR-21-5p, and bta-miR-141, which can be used as biomarkers for the identification of pregnancy problems (Ali et al., 2021). The expression of miR-146b is upregulated during pregnancy. The concentration of this miRNA is greater in luminal progenitors as compared to basal/stem cells. Mammary epithelial cell differentiation is similarly impacted by it (Dysin et al., 2021). Mice have been found to express miR-146b, notably in luminal progenitors, indicating that it plays a role in the development of mammary stem cells. Similar to previous research, we found that bta-miR-146b, bta-miR-27b, 423-3p, bta-miR-21-5p, and bta-miR-141 were upregulated during the early days of pregnancy in buffalo. The ROC analysis indicated that the accuracies of the miRNAs which were upregulated in this study were 60%–80%. Protein-based assays, such as PAG-based ELISA, have shown 93% accuracy on the 30^th^ day of pregnancy, whereas ISG15-based ELISA has revealed 80% accuracy on the 18^th^ day of pregnancy (Green et al., 2005; Batra et al., 2018a; Batra et al., 2018b). ROC analyses revealed that individual miR-26a in cattle could predict pregnancy outcome with much lower accuracy than WBC ISG15 on 18^th^ day (Tzelos et al., 2023). Because of environmental risk factors, viral infections, and the high likelihood of early embryonic death during the implantation stage, these miRNAs also lack specificity (Takino et al., 2016; Alhussien et al., 2018). Therefore, a panel of miRNAs can act as a suitable biomarker for early pregnancy diagnosis in buffalo.

## 5 Conclusion

In conclusion, the current study profiled the whole miRNAs population in the maternal blood of buffalo during the initial stages of pregnancy. Ten unique miRNAs that can distinguish between pregnant and non-pregnant buffaloes on or before the 18^th^ day of pregnancy were identified in this investigation. An excellent plasma biomarker for diagnosing early pregnancy in buffaloes is miR-181a and miR-486, which are especially prevalent in the maternal blood of buffaloes on day 18^th^ of pregnancy. Also, it would be easier to identify rebreeding in non-pregnant animals if it were confirmed that their blood miR-191 and miR-let-7f quantity was lower than that of pregnant animals on 18^th^ day. The biological functions of the miR-s linked to pregnancy need to be clarified in future functional validation studies before pregnancy diagnostic kits can be commercially introduced.

## Data Availability

The datasets presented in this study can be found in online repositories. The names of the repository/repositories and accession number(s) can be found below: https://www.ncbi.nlm.nih.gov/, PRJNA1091188, SAMN40589300, SAMN40589301, SAMN40589302, SAMN40589303, SAMN40589304, SAMN40589305, SAMN40589306, SAMN40589307, SAMN40589308, SAMN40589309, SAMN40589310, SAMN40589311, SAMN40589312, SAMN40589313, SAMN40589314, SAMN40589315, SAMN40589316, SAMN40589317.

## References

[B1] Acuña-González R. J. Olvera-Valencia M. López-Canales J. S. Lozano-Cuenca J. Osorio-Caballero M. Flores-Herrera H. (2021). MiR-191-5p is upregulated in culture media of implanted human embryo on day fifth of development. Reprod. Biol. Endocrinol. 19 (1), 109. 10.1186/s12958-021-00786-1 34256783 PMC8278618

[B2] Alhussien M. N. Kamboj A. Aljader M. A. Panda B. S. K. Yadav M. L. Sharma L. (2018). Effect of tropical thermal stress on peri-implantation immune responses in cows. Theriogenology 114, 149–158. 10.1016/j.theriogenology.2018.03.036 29625402

[B3] Ali A. Hadlich F. Abbas M. W. Iqbal M. A. Tesfaye D. Bouma G. J. (2021). MicroRNA-mRNA networks in pregnancy complications: a comprehensive downstream analysis of potential biomarkers. Int. J. Mol. Sci. 22 (5), 2313. 10.3390/ijms22052313 33669156 PMC7956714

[B4] Andrews S., (2010). FASTQC: a quality control tool for high throughput sequence data. Available online at: http://www.bioinformatics.babraham.ac.uk/projects/fastqc.

[B5] Balaguer N. Moreno I. Herrero M. Gonzáléz-Monfort M. Vilella F. Simón C. (2019). MicroRNA-30d deficiency during preconception affects endometrial receptivity by decreasing implantation rates and impairing fetal growth. Am. J. Obstet. Gynecol. 221 (1), 46.e1–46. 10.1016/j.ajog.2019.02.047 30826341

[B6] Barile V. L. Menchetti L. Casano A. B. Brecchia G. Melo de Sousa N. Zelli R. (2021). Approaches to identify pregnancy failure in Buffalo cows. Animals 11 (2), 487. 10.3390/ani11020487 33673362 PMC7917614

[B7] Batra K. Kumar A. Maan S. Kumar V. Kumari R. Nanda T. (2018a). Recombinant interferon stimulated protein 15 (rISG15) as a molecular marker for detection of early pregnancy in Bubalus bubalis. Anim. Reprod. Sci. 197, 106–116. 10.1016/j.anireprosci.2018.08.018 30145042

[B8] Batra K. Nanda T. Kumar A. Kumar V. Gopal G. J. Maan S. (2018b). Molecular cloning and expression kinetics of serum interferon stimulated gene for early pregnancy detection. Indian J. Anim. Res. 53 (9), 1129–1134. 10.18805/ijar.b-3636

[B9] Battaglia R. Palini S. Vento M. E. La Ferlita A. Lo F. Caroppo M. J. (2019). Identification of extracellular vesicles and characterization of miRNA expression profiles in human blastocoel fluid. Sci. Rep. 9 (1), 84. 10.1038/s41598-018-36452-7 30643155 PMC6331601

[B10] de Ávila A. C. F. C. M. Bridi A. Andrade G. M. Del Collado M. Sangalli J. R. Nociti R. P. (2020). Estrous cycle impacts microRNA content in extracellular vesicles that modulate bovine cumulus cell transcripts during *in vitro* maturation. Biol. Reprod. 102 (2), 362–375. 10.1093/biolre/ioz177 31504242

[B11] de Carvalho N. A. Soares J. G. Baruselli P. S. (2016). Strategies to overcome seasonal anestrus in water buffalo. Theriogenology 86 (1), 200–206. 10.1016/j.theriogenology.2016.04.032 27157389

[B12] Dysin A. P. Barkova O. Y. Pozovnikova M. V. (2021). The role of microRNAs in the mammary gland development, health, and function of cattle, goats, and sheep. Non-coding RNA 7 (4), 78. 10.3390/ncrna7040078 34940759 PMC8708473

[B13] Fiandanese N. Viglino A. Strozzi F. Stella A. Williams J. L. Lonergan P. (2015). 71 CIRCULATING microRNAs AS POTENTIAL BIOMARKERS OF EARLY PREGNANCY IN HIGH-PRODUCING DAIRY COWS. Reprod. Fertil. Dev. 28, 165. 10.1071/rdv28n2ab71

[B14] Gebremedhn S. Salilew-Wondim D. Hoelker M. Held-Hoelker E. Neuhoff C. Tholen E. (2018). Exploring maternal serum microRNAs during early pregnancy in cattle. Theriogenology 121, 196–203. 10.1016/j.theriogenology.2018.08.020 30172131

[B15] Green J. A. Parks T. E. Avalle M. P. Telugu B. P. McLain A. L. Peterson A. J. (2005). The establishment of an ELISA for the detection of pregnancy-associated glycoproteins (PAGs) in the serum of pregnant cows and heifers. Theriogenology 63 (5), 1481–1503. 10.1016/j.theriogenology.2004.07.011 15725453

[B16] Gross N. Kropp J. Khatib H. (2017). MicroRNA signaling in embryo development. Biology 6 (3), 34. 10.3390/biology6030034 28906477 PMC5617922

[B17] Guelfi G. Stefanetti V. De Luca S. Giontella A. Barile V. L. Barbato O. (2017). Serum microRNAs in buffalo cows: potential biomarkers of pregnancy. Res. Vet. Sci. 115, 294–300. 10.1016/j.rvsc.2017.06.001 28628844

[B18] Ioannidis J. Donadeu F. X. (2016). Circulating miRNA signatures of early pregnancy in cattle. BMC genomics 17, 184. 10.1186/s12864-016-2529-1 26939708 PMC4778341

[B19] Ioannidis J. Donadeu F. X. (2017). Changes in circulating microRNA levels can be identified as early as day 8 of pregnancy in cattle. PloS one 12 (4), e0174892. 10.1371/journal.pone.0174892 28380001 PMC5381804

[B20] Legare C. Clement A. A. Desgagné V. Thibeault K. White F. Guay S. P. (2022). Human plasma pregnancy-associated miRNAs and their temporal variation within the first trimester of pregnancy. Reprod. Biol. Endocrinol. 20 (1), 14. 10.1186/s12958-021-00883-1 35031065 PMC8759232

[B21] Lim H. J. Kim H. J. Lee J. H. Lim D. H. Son J. K. Kim E. T. (2021). Identification of plasma miRNA biomarkers for pregnancy detection in dairy cattle. J. Anim. Reprod. Biotechnol. 36 (1), 35–44. 10.12750/jarb.36.1.35

[B22] Ma R. Liang Z. Shi X. Xu L. Li X. Wu J. (2021). Exosomal miR-486-5p derived from human placental microvascular endothelial cells regulates proliferation and invasion of trophoblasts via targeting IGF1. Hum. Cell 34 (5), 1310–1323. 10.1007/s13577-021-00543-x 33977502 PMC8338855

[B23] Markkandan K. Lim H. J. Lee D. J. Shin I. G. Yoo S. I. Dang C. (2018). Data on transcriptome profiling of circulating microRNAs in dairy cattle. Data brief 21, 775–778. 10.1016/j.dib.2018.09.038 30417039 PMC6216090

[B24] Ono K. Okamoto S. Ninomiya C. Toji N. Kanazawa T. Ishiguro-Oonuma T. (2022). Analysis of circulating microRNA during early gestation in Japanese black cattle. Domest. Anim. Endocrinol. 79, 106706. 10.1016/j.domaniend.2021.106706 34973621

[B25] Ospina-Prieto S. Chaiwangyen W. Herrmann J. Groten T. Schleussner E. Markert U. R. (2016). MicroRNA-141 is upregulated in preeclamptic placentae and regulates trophoblast invasion and intercellular communication. Transl. Res. 172, 61–72. 10.1016/j.trsl.2016.02.012 27012474

[B26] Paulson E. E. Fishman E. L. Schultz R. M. Ross P. J. (2022). Embryonic microRNAs are essential for bovine preimplantation embryo development. Proc. Natl. Acad. Sci. U. S. A. 119 (45), e2212942119. 10.1073/pnas.2212942119 36322738 PMC9659414

[B27] Petrocchi Jasinski F. Evangelista C. Basiricò L. Bernabucci U. (2023). Responses of dairy Buffalo to heat stress conditions and mitigation strategies: a review. Animals 13 (7), 1260. 10.3390/ani13071260 37048516 PMC10093017

[B28] Pohler K. G. Green J. A. Moley L. A. Gunewardena S. Hung W. T. Payton R. R. (2017). Circulating microRNA as candidates for early embryonic viability in cattle. Mol. Reprod. Dev. 84 (8), 731–743. 10.1002/mrd.22856 28643872 PMC5580359

[B29] Reza A. M. M. T. Choi Y. J. Han S. G. Song H. Park C. Hong K. (2019). Roles of microRNAs in mammalian reproduction: from the commitment of germ cells to peri-implantation embryos. Biol. Rev. Camb Philos. Soc. 94 (2), 415–438. 10.1111/brv.12459 30151880 PMC7379200

[B30] Sarwalia P. Raza M. Soni A. Dubey P. Chandel R. Kumar R. (2021). Establishment of repertoire of placentome-associated MicroRNAs and their appearance in blood plasma could identify early establishment of pregnancy in Buffalo (*Bubalus bubalis*). Front. Cell. Dev. Biol. 9, 673765. 10.3389/fcell.2021.673765 34513824 PMC8427669

[B31] Schmittgen T. D. Livak K. J. (2008). Analyzing real-time PCR data by the comparative C(T) method. Nat. Protoc. 3 (6), 1101–1108. 10.1038/nprot.2008.73 18546601

[B32] Takino T. Okamura T. Ando T. Hagiwara K. (2016). Change in the responsiveness of interferon-stimulated genes during early pregnancy in cows with Borna virus-1 infection. BMC Vet. Res. 12 (1), 253. 10.1186/s12917-016-0883-5 27842550 PMC5109691

[B33] Tzelos T. Lee S. Pegg A. Donadeu F. X. (2023). Association between blood miR-26a levels following artificial insemination, and pregnancy outcome in dairy cattle. PloS one 18 (8), e0289342. 10.1371/journal.pone.0289342 37566616 PMC10420342

[B34] Wang D. Zhang Z. O'Loughlin E. Lee T. Houel S. O'Carroll D. (2012). Quantitative functions of Argonaute proteins in mammalian development. Genes Dev. 26 (7), 693–704. 10.1101/gad.182758.111 22474261 PMC3323880

[B35] Zhai Y. Shi Q. Chu Q. Chen F. Feng Y. Zhang Z. (2022). miRNA profiling in intrauterine exosomes of pregnant cattle on day 7. Front. Vet. Sci. 9, 1078394. 10.3389/fvets.2022.1078394 36605764 PMC9810022

[B36] Zhang Q. Zhang H. Jiang Y. Xue B. Diao Z. Ding L. (2015). MicroRNA-181a is involved in the regulation of human endometrial stromal cell decidualization by inhibiting Krüppel-like factor 12. Reprod. Biol. Endocrinol. 13, 23. 10.1186/s12958-015-0019-y 25889210 PMC4379545

[B37] Zhao H. Wang L. Wang Y. (2021). Circulating microRNAs as candidate biomarkers for the ovarian response during *in vitro* fertilization. Medicine 100 (6), e24612. 10.1097/MD.0000000000024612 33578569 PMC7886401

